# Competency assessment of an undergraduate program using a third-party, objective pre-post examination

**DOI:** 10.1186/s12909-020-02448-z

**Published:** 2021-01-06

**Authors:** Lawrence Fulton, Cristian Lieneck, Zo Ramamonjiarivelo, Clemens Scott Kruse, Matthew S. Brooks

**Affiliations:** Department of Health Administration, 601 University Drive, Texas State University, San Marcos, TX 78666 USA

**Keywords:** Competency-based medical education (CBME), Competency assessment, Pre-post testing, Quasi-experimental, Peregrine testing

## Abstract

**Background:**

Assessing competencies or program learning outcomes in educational programs is often a leadership challenge. This case study reports medical education program’s efforts to document undergraduate competency attainment using a pre-post, third-party, objective testing service that allows for inter-university comparison, a testing service that is being adopted by some certification and accrediting bodies.

**Methods:**

Students completed a pre-test after program acceptance and a post-test at the end of the last didactic semester (1.5 years later) just prior to their required internships. Scores and subscores were evaluated using t-tests (Holm-adjusted *p*-values). MANOVA models of sub-competency difference scores were also evaluated.

**Results:**

Results indicate competency improvement for each of the 12 areas based on the *n* = 55 student sample, (*p* < .001 for all scores). These improvements were independent of ethnicity, age, gender, and grades. The average student improved by 12.85 points (95% CI of 10.52 to 15.18) with the largest improvements in strategic planning and leadership competency areas (21.30 and 18.33 percentage points, respectively).

**Conclusions:**

The third-party pre-post has some face validity given that student performance improved after completing a related curriculum as would be expected. Congruent with earlier studies, we find that repeated testing helps document competency attainment and that a single method for assessment is insufficient. We further document limitations of this 3d-party exam.

## Highlights

- Assessing the efficacy of undergraduate program success in achieving program goals is difficult
- An undergraduate medical program adopted Internet-based pre-post competency testing as part of assessment
- Paired tests with Holm-adjusted *p*-values show improvement in all competency areas, confirming program efficacy
- MANOVA of difference scores as a function of demographics and grades shows effects only from differencing
- This type of pre-post testing is important to verify the efficacy of the curriculum intervention
- The 3d-party tool provides inter-university comparisons but requires improvement

## Background

Competency-based medical education (CBME) is the standard for many education programs in the medical field [1]. In the disciplines of healthcare administration, management, and leadership (HAML), CBME is required for undergraduate programs seeking certification from the Association of University Programs in Health Administration [2] as well as for graduate programs applying for the Commission on Accreditation of Healthcare Management Education accreditation [3]. Further, HAML programs are often coupled with other medical education programs [4, 5], and it is important that students attain the associated competencies advertised. HAML programs are ubiquitous, existing in health science centers, medical schools, schools of allied health, colleges of health professions, and business schools, and the importance of the competency focus in these programs has been well established [6, 7]. Despite the large number of HAML programs and globalization of the medical sector, there are no required national or international competency assessments for these programs [8]. Further, programs need not assess competencies at all if they do not seek certification or accreditation from a separate agency.

The primary purpose of a CBME-based program is to produce graduates who possess specific competencies required of the profession. Using the definition of Gervais, competency-based education (CBE) is outcome-based and incorporates delivery and assessment modes for evaluating “mastery of learning by students through their demonstration of knowledge, attitude, values, skills, and behaviors required for the degree sought” [9]. Healthcare leaders expect graduates with HAML-related degrees to have obtained the requisite competencies for management of complex organizations [10, 11]. Competencies are defined as “a cluster of related knowledge, skills, and attitudes that: 1) affect a major part of one’s job, 2) are correlated with performance on the job, 3) can be measured against accepted standards, and 4) can be improved training and development” [12].

Assessing CBME for medical education programs is both non-trivial and often non-standardized. Some research has suggested that a systems perspective is necessary and that any evaluation should leverage academic advisors [13]. Other research has focused on the value of a programmatic approach [14, 15]. Although recommendations regarding CBME exist, little empirical research about programmatic assessment exists [16].

There are exceptions, however. For example, Germany is leading an effort to build valid competency assessment tools for engineering, economics, social sciences, educational science, psychology, and teacher training. This effort is multi-institutional, international, and collaborative [17]. Part of this effort combined the Mexican Examen General para el Egreso de la Licenciatura (EGEL) and the American Test of Understanding in College Economics (TUCE IV) by the Council for Economic Education (CEE) into a single measurement instrument that was validated comprehensively [18]. Germany has also adopted the Masterplan Medizinstudium 2020 to shift its medical licensing examination model to a competency-based model [19].

Also, there are efforts for longitudinal assessment of competency attainment of undergraduate students in some organizations. A specific example is the Valid Assessment of Learning in Undergraduate Education (VALUE) program implemented by Queen’s University. VALUE evaluates longitudinally student attainment of competencies as well as critical thinking, cognitive skills, lifelong learning, problem solving, and transferrable learning orientations for bachelor of arts, bachelor of computing, bachelor of education, bachelor of nursing science, and bachelor of science in engineering programs. While not specifically related to HAML, the initiative demonstrates a focus on quantification of competency attainment by students for system-wide educational improvement [20].

### CBME methods in HAML programs

Prior HAML-program studies have examined the effectiveness of CBME from different perspectives using survey instruments. For instance, one study assessed the effectiveness of competency-based programs by comparing survey ratings from preceptors with residents/fellows competency self-ratings and they found that residents/fellows tended to rate themselves higher than the preceptors [21]. Bradley et al., (2008) used a cross-sectional analysis to assess students’ competency development by comparing data from self-rated competencies of entering students with self-ratings of returning students and self-ratings of new graduates; they found that new graduates had higher self-ratings than entering students and returning students had higher self-ratings than entering students [22]. Friedman and Frogner (2010) conducted a survey of healthcare leaders who perceived themselves as “early careerists” to self-rate their competency levels and rate the competency levels of new graduates. They found that healthcare leaders with MHA degrees tended to rate themselves higher and rate new graduates lower, compared to healthcare leaders without MHA degrees [23]. Lomperis et al., (2012) used student surveys to measure competency attainment, but this study took a different approach, implementing an oral comprehensive exam based on case studies [11].

While the approaches used by the studies may be appropriate for qualitative measurement of competency attainment, they are non-standardized, non-objective, and non-a priori. Students may arrive with high-level competencies, so the effectiveness of the program cannot be known without time-sequenced assessments or (at least) pre-post testing and evaluation. At a minimum, both pre and post evaluations of competencies are required to determine the efficacy of a program curriculum “treatment.” Further, preceptor and self-assessments as well as oral examinations of competency attainment may be biased due to the halo effect as well as other sources [24]. Exclusive use of self and preceptor assessment results in an inestimable amount of bias and thus confounds an understanding of program improvement. As suggested by previous research, multiple methods should be used for assessment of competency attainment, including standardized testing [20].

There appears to be little standardization of competency-assessment in HAML programs. We found one HAML study (a student self-assessment for an interdisciplinary leadership program) that used any pre-post analysis to determine whether students improved [25]. To address this concern certification and accrediting bodies for HAML (e.g.,, the Association of University Programs in Healthcare or AUPHA and the Commission on Accreditation of Healthcare Management Education or CAHME) have sought out corporate partners for competency assessment testing [26, 27].

### Purpose and significance

The purpose of this study was to assess the effectiveness of a program-based CBME for an undergraduate HAML program (health administration) in a college of health professions using testing provided by a third party, Peregrine Academic Services (PAS), based on program competencies. The intent was to evaluate whether program performance might be assessed at the student level for individual competency attainment, at the program level for quality improvement, and at the national level for benchmarking. This type of multi-dimensional assessment is required for certification of many programs. Limitations of this test are evaluated in the discussion. Since a pre-post difference should be expected, this was the first attempt at evaluating some face validity of the proposed test as well.

The significance of this study involves program certification and performance improvement. Regardless of program type, certification or accreditation typically requires assessment of student competency attainment as well as program assessment for quality improvement purposes. Grades in courses do not effectively translate to individual competencies, as a single course might address small portions of various competencies. For example, the ability to communicate is a competency often found in multiple courses, yet the grade for those courses are likely not specific to the communication competency. Using PAS competency assessment testing based on AUPHA requirements allowed for one objective assessment. Coupled with preceptor and individual assessments, a 360-degree view of a program’s strengths and weaknesses might better be obtained.

### Research question and associated hypotheses

The primary research question investigates whether pre-post, third-party objective testing provides evidence that the medical education programs in the study effectively increases competency performance scores (and associated percentile rankings) as well as scores for each of the subordinate competencies. The subordinate competencies that compose this score are defined by the Healthcare Leadership Alliance [28] and measured by PAS pre-post testing [29] and are shown in Table 1.

**Table 1:** Overall descriptive statistics for the pre-test, the post-test, and post-test minus pre-test (difference scores)

	Means			Medians			SD		
	Pre	Post	Delta	Pre	Post	Delta	Pre	Post	Delta
Strategic Planning and Marketing	56.1	77.4	21.3	60	80	20	18	12	19.8
Leadership Skills and Behavior	58.7	77.0	18.3	60	80	20	15.7	15.1	21.0
General Management	59.3	77.2	18.0	60	80	20	16.1	11.6	20.8
Financial Management	55.9	73.5	17.6	50	70	20	18.2	15.2	22.6
Quality Improvement	58.0	73.0	15.0	60	70	20	18.3	13.8	20.4
Community and the Environment	53.1	68.1	15.0	50	70	15	16.6	16.3	22.9
Healthcare Personnel	64.6	79.4	14.8	60	80	10	14.5	13.1	21.2
Healthcare Systems & Organizations	53.3	68.0	14.6	50	70	15	19.2	16.3	26.3
Legal Environment of Healthcare	61.1	74.6	13.5	60	80	10	16.2	17.3	23.8
Organizational Climate and Culture	63.9	75.7	11.9	60	80	10	17	16.2	19.4
Managing Change	61.5	70.6	9.07	60	70	10	13	13.7	19.6
Information Management	58.3	66.7	8.33	60	70	10	18.3	12.3	22.0
Final Score	58.7	71.5	12.9	60	72	12.4	7.92	5.13	8.54
Percentile Rank	58.7	80.4	21.7	63	84	20	22.9	15.7	25.5

This study is significant in that it evaluates pre-post performance of an entire program in developing student competencies using PAS as defined by HLC. Since PAS is a platform that AUPHA is considering for undergraduate program certification assessment, it is important to assess its prima facia validity, strengths, and weaknesses. On a larger scale, “assessing an assessment instrument” is important for the larger medical education community. While one would expect increases in performance after a program’s worth of instruction, it is possible that these increases might be despite the assessment instrument rather than because of it. This study also illustrates the importance of using metrics to drive program direction.

## Methods

### Setting

The setting for this study is a large, public university in Texas that is Southern Association of Colleges and Schools (SACS) accredited with a health administration undergraduate program that is AUPHA certified and located in a college of health professions. The study program has up to 160 students at any given time. The university is a Hispanic-Serving Institution (HIS-greater than 25% Hispanic students), and the program itself has closer to 30–50% Hispanic students at any given time.

### Instrument

PAS provides on online test based on competencies for health administration, business administration, and other disciplines. PAS individual test results include the overall test score and percentile ranking based on other academic programs. PAS also provides scores for each assessed competency area.. For the study program students, the testing seeks to measure competency attainment. Notable exceptions omitted from this study are the quantitative analysis competency and communication (measured internally in several courses). (The quantitative analysis competency was added from PAS’s business school components to the study university’s version of the test in late 2019, so no pre-test scores are yet available. The communication competency is assessed in several classes by faculty-designed, writing or speaking-intensive projects.) The PAS test questions themselves are multiple choice. One should note that PAS testing is only one of 34 assessments used by the program to assess competencies and program outcomes. A complete list of program and student learning objective assessments (as well as the analysis) is available online: https://rpubs.com/R-Minator/PASTesting.

While PAS questions evaluate largely at the “knowledge” level, some of the questions assess at the application level of Bloom’s taxonomy [30]. PAS is a corporate partner of the Association of University Programs in Healthcare [27], and AUPHA is adopting PAS testing to evaluate undergraduate programs across the nation. The program needed to assess the efficacy of the certification examination in documenting student performance improvements as part of an assessment of face validity.

The PAS assessment as implemented in this setting provides students 10 random questions in each of the formally assessed competency areas (120 question). For the pre-test, students receive three minutes per question on average. For the post-test (given at least 1.5 years after the pre-test), two hours are provided. Extending the test beyond two minutes per question has shown no improvement in individual performance within the program; diminishing returns are experienced. The undergraduate medical program funds the cost of each test. The testing is not currently computer adaptive, and the questions are not assigned a difficulty rating (see the detailed discussion in the limitations section).

### Sample

The study university has been using Peregrine testing in Fall 2018 and implemented pre-post testing in January 2019. Since the start of testing, students have taken 243 valid tests, and about a quarter of them completed both the pre-test and the post-test (*n* = 54). Some students completed only the post-test during early adoption, while others have completed only the pre-test pending matriculation. These students comprise the cohort-based, sequential sample for this study. Students must take the pre-test as a condition for entering the program and must pass the post-test based on faculty-determined criteria prior to their internships. Currently, students must obtain overall scores above the 50th percentile to pass the examination; however, that is being adjusted upward to 50th percentile for each of the 12 measured competencies. The Texas State University Institutional Review Board deemed this research exempt (IRB application 7234).

### Data analysis

With multiple dependent variables included in this analysis, we ran t-tests with Holm-adjusted *p*-values to account for familywise error [31]. Since the data are quantitative continuous and the sample size is sufficiently large to assume normality of means, the t-test with familywise error correction are a reasonable choice. Additionally, we ran multiple analysis of variance on the pre-post difference scores (thus accounting for repeated measures) for all sub-competencies to evaluate the effects of gender, ethnicity, and grade point average (GPA). Analyses were conducted using R Statistical Software [32].

## Results

### Descriptive statistics

**Fig. 1:**
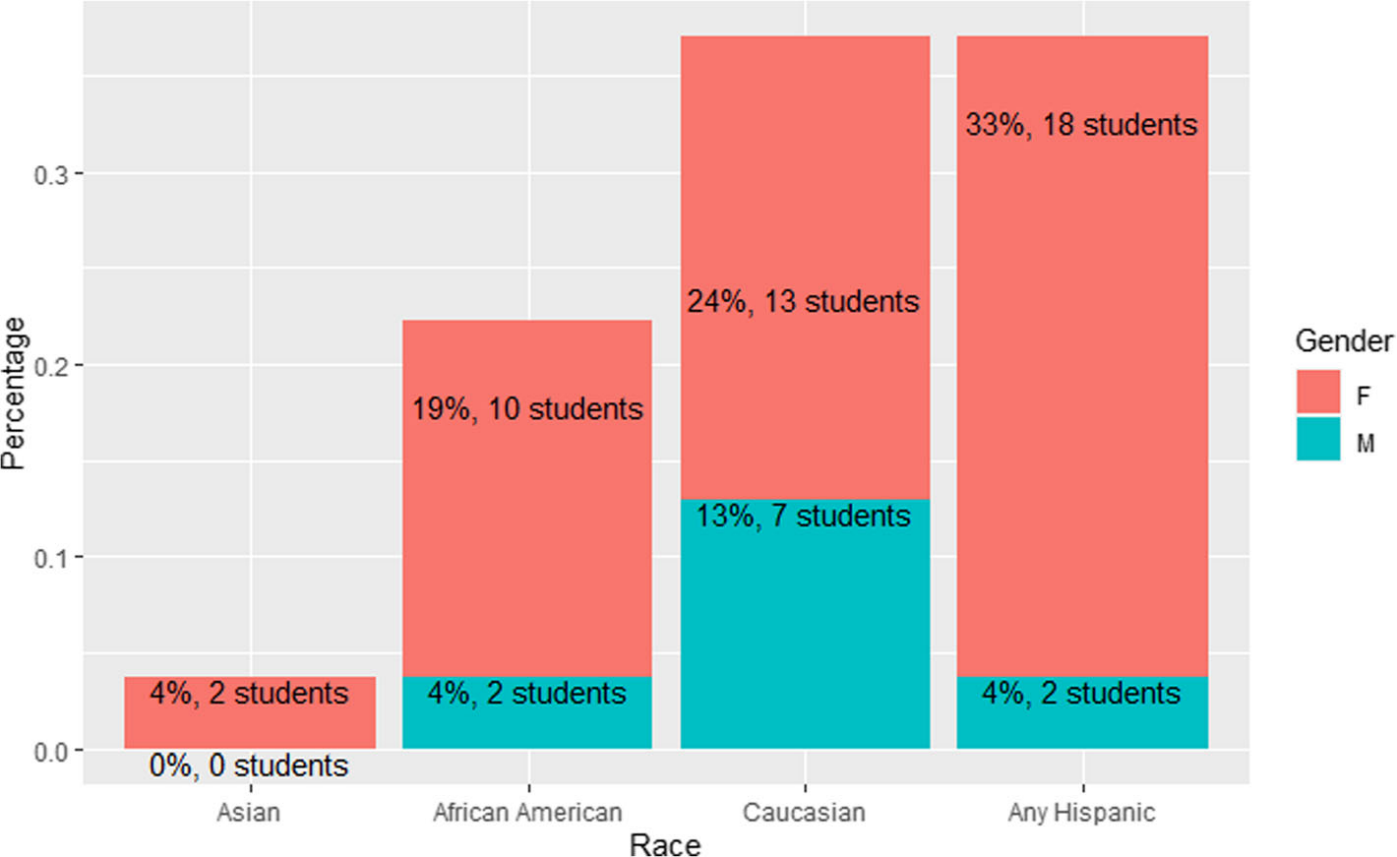
Distribution of students by race and gender

All statistical analyses are available here: https://rpubs.com/R-Minator/PASTesting. The base demographics of the sample are shown in Fig. 1. Overall, 37% of the population were classified by the university as Hispanics, 37% were Caucasian, 22% were African American, and 4% were Asian. Further, the majority of students (79.6%) were female. The study university represents a regionally appropriate diverse student body.

**Fig. 2:**
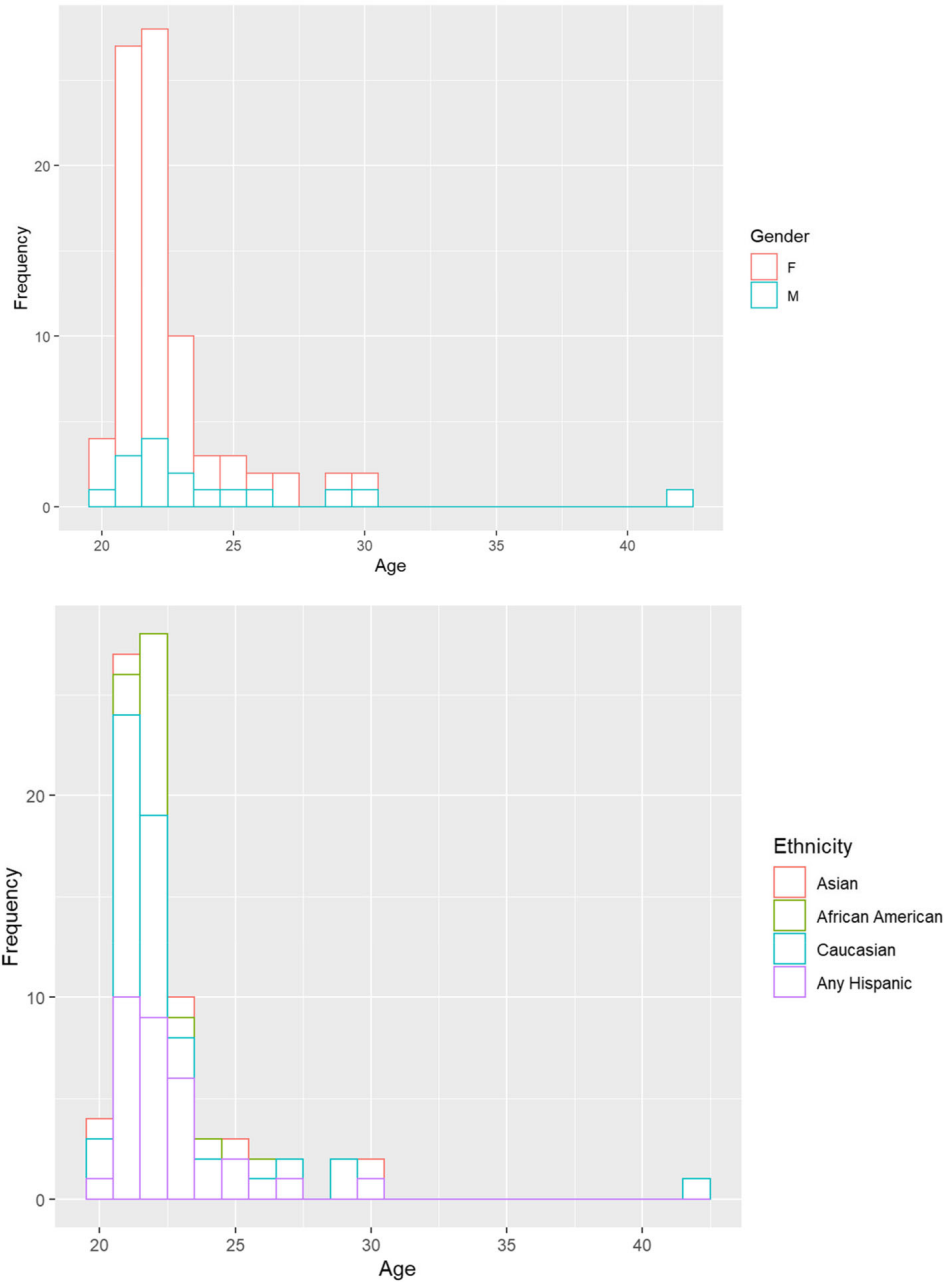
Distribution of age by gender (top) and by ethnicity / race (bottom)

The mean age of students in the sample was 22.96 years old (median = 22, SD = 3.30). The three outliers depicted in Fig. 2 were all males (a minority) with prior workforce experience. No African American students were older than 26.

**Fig. 3:**
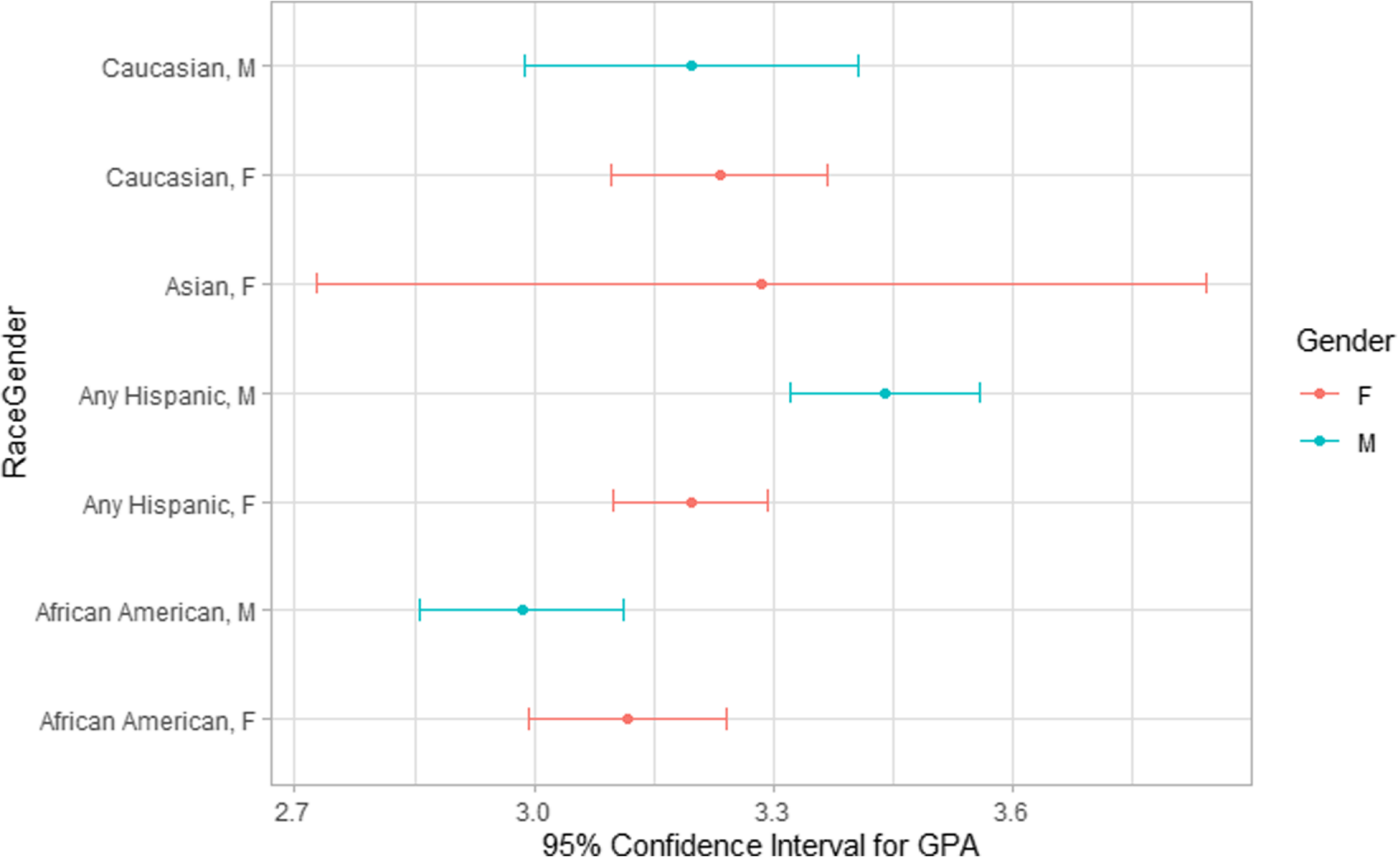
95% confidence intervals for GPA based on gender and race

The mean GPA for students in the study was 3.20 (median 3.19, SD = .23). Since the minimum requirement for program admission is 2.75 with the follow-on requirement that 3.0 or better must be maintained, these values seem appropriate. The GPA did not statistically differ based on ethnicity or gender as shown in Fig. 3.

The average days between the pre-test and post-test was 435.54 (median = 430, SD = 44.18). This time between tests mitigate any concern that there was test improvement based solely on content learned from the pre-test.

**Fig. 4:**
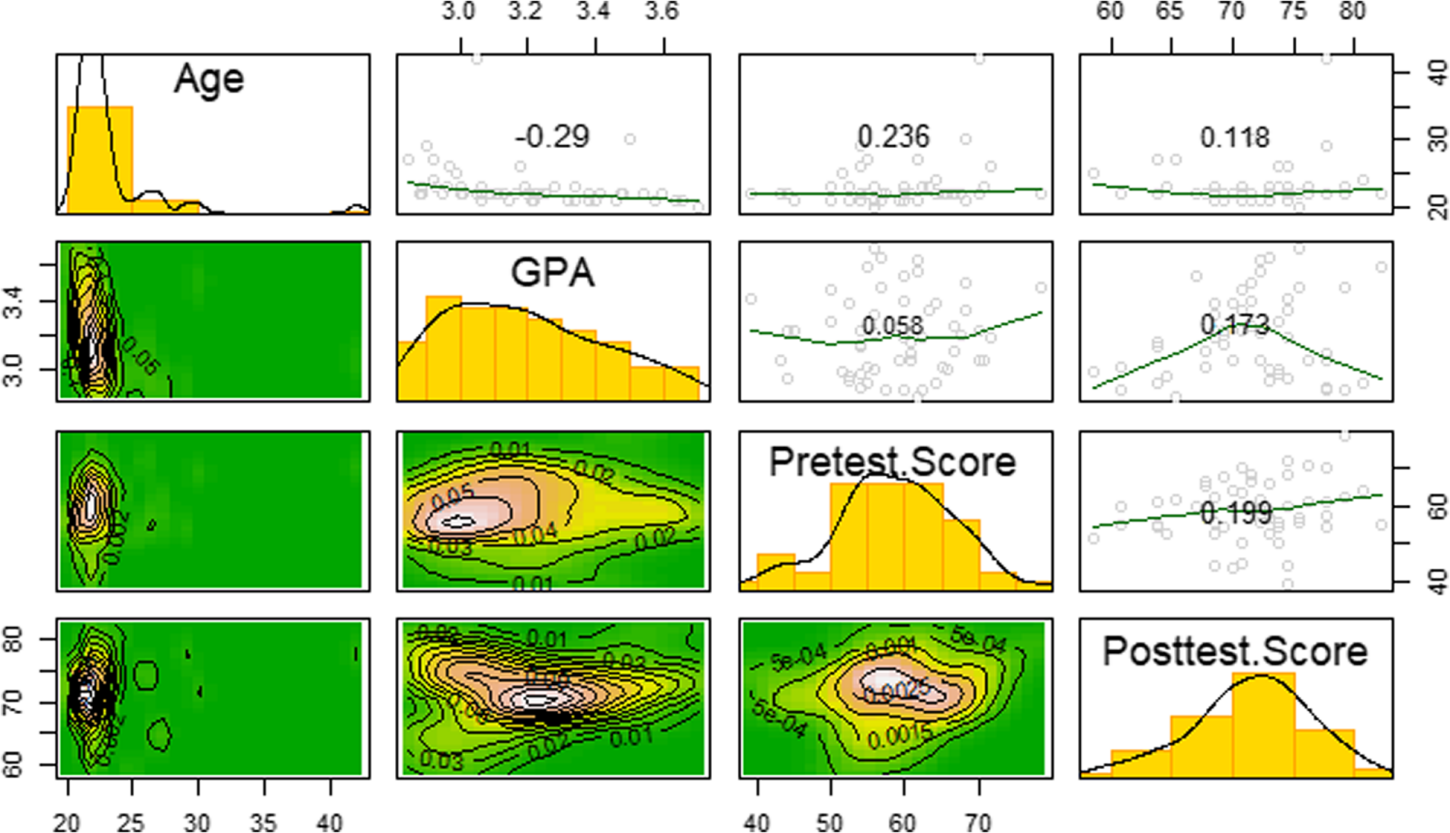
Scatterplot matrix with bivariate contour plots, histograms, kernel density estimates, and loess curve estimates of the bivariate scatterplot relationships

The relationships among age, GPA, pre-test scores, and post-test scores are not very revealing. Only GPA and age are statistically related, and that relationship is slightly negative (r = − 0.29). Age and pre-test scores are weakly and positively correlated (r = .236), but the evidence is weak supporting this relationship (*p* = .09). Most interestingly, the pre-test performance is not related to the post-test performance (ρ = .199) when not accounting for individual test-taking performance. Figure 4 provides an enhanced scatterplot matrix with histograms and kernel density estimates on the diagonal, contour plots on the lower diagonal, and scatterplots with correlations on the upper diagonal.

**Fig. 5:**
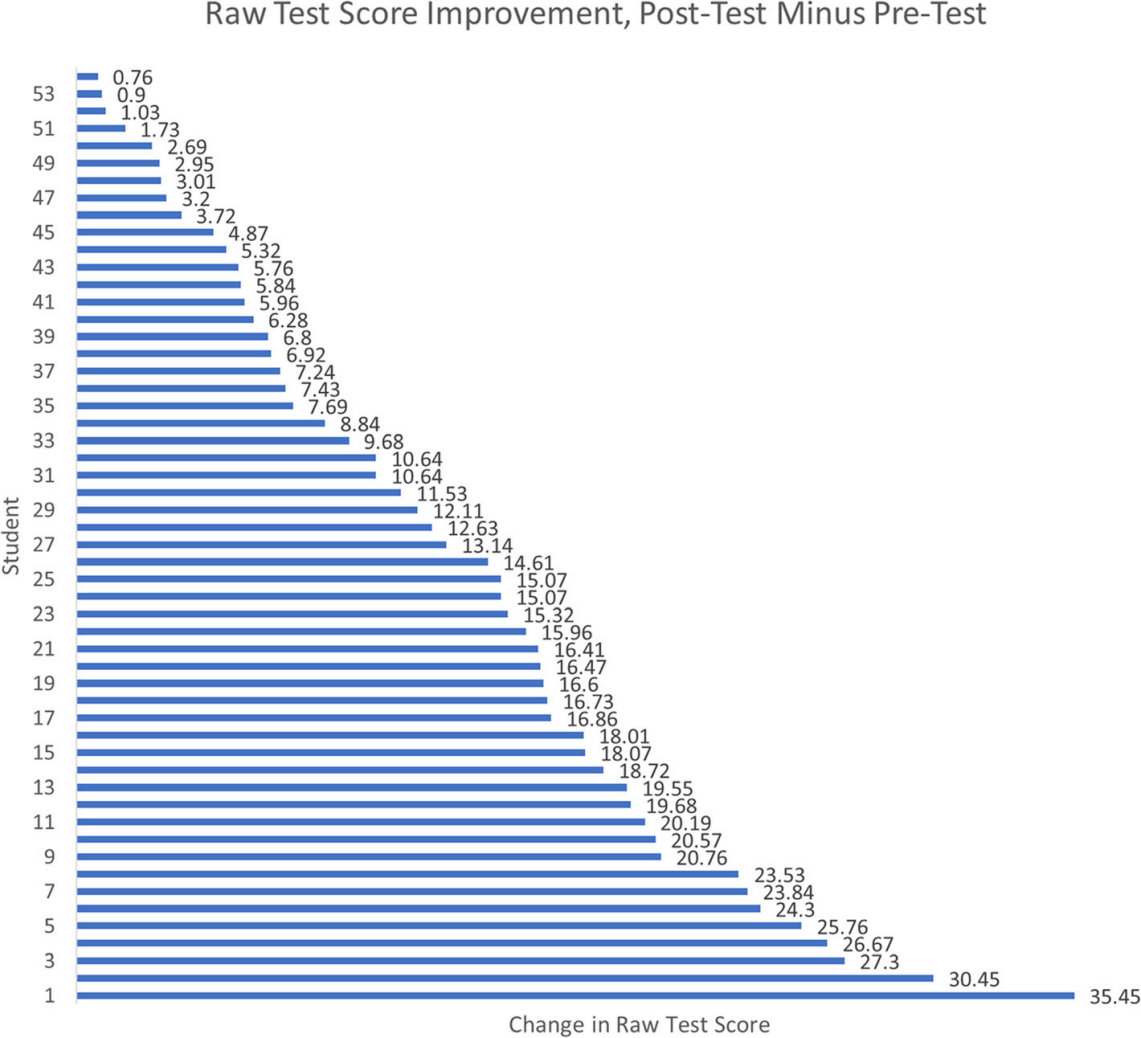
Raw test score improvements

Table 1 provides the descriptive statistics for the *n* = 54 sample pre-test, post-test, and difference scores overall and by subordinate competency. Also provided is the completion time (which on average increased from pre-test to post-test) and the percentile ranking relative to undergraduates from all universities who took the post-test. The “average” student increased 12.85 points, from 58.65 to 71.51 in final score. The medians were comparable indicating little skew. The largest gains were in the areas of strategic planning and marketing, leadership skills and behavior, general management, and financial management (median gains of 20 points each). Little overall improvement was seen in the legal environment of healthcare, organizational climate and culture, managing change, and information management (median gains of 10 points each). Figure 5 depicts the individual improvement in raw test scores.

**Fig. 6:**
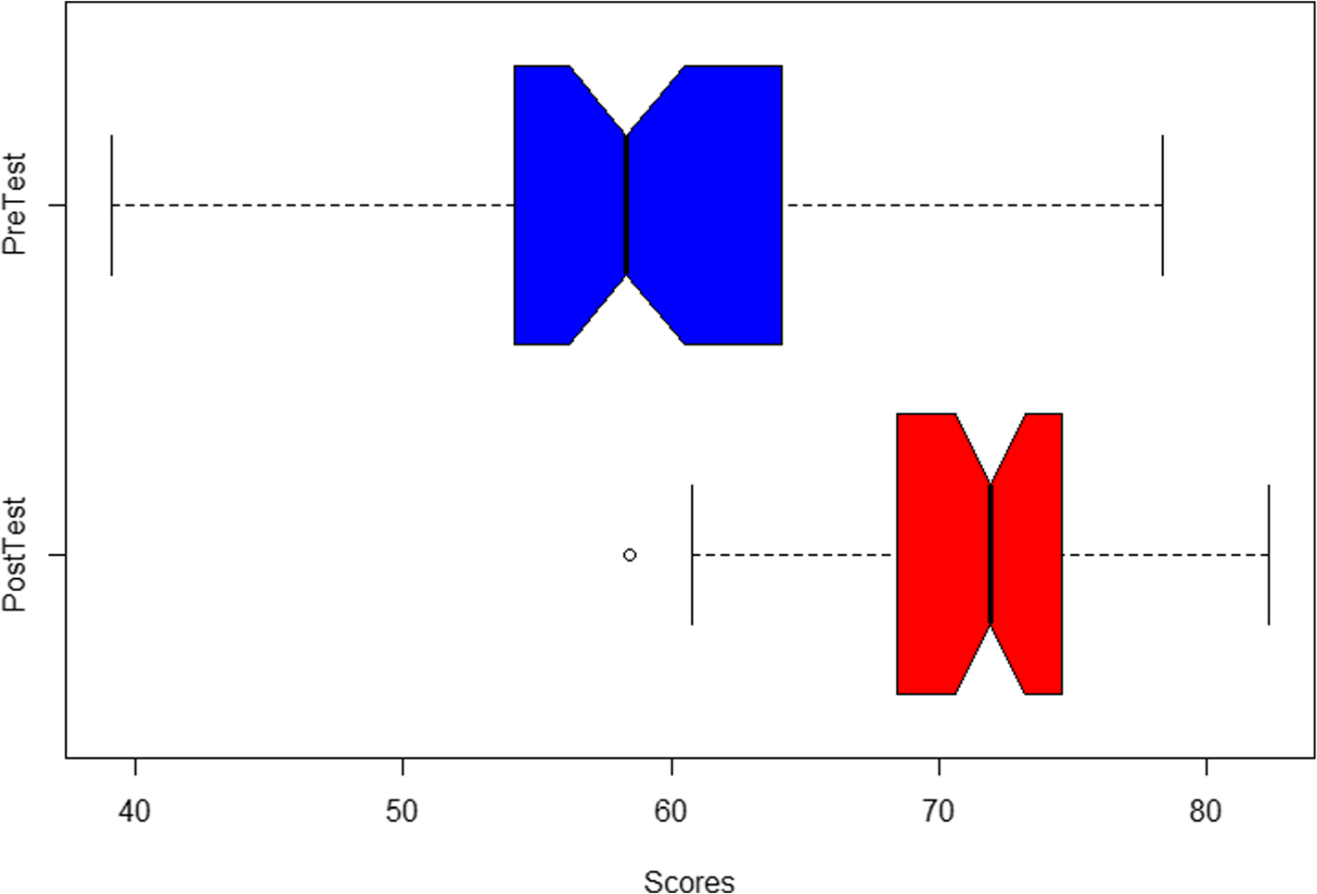
Pre-post notched boxplots

Fig. 6 shows notched boxplots of the pre and post-test scores. Notched boxplots provide a graphical median test of the distributions. If the notched area of one distribution does not intersect the notched area of the other distribution, then the medians are different at the α = .05 level. The graph shows that the unpaired distributions are different; however, this graphical analysis does not account for individual capability.

**Fig. 7:**
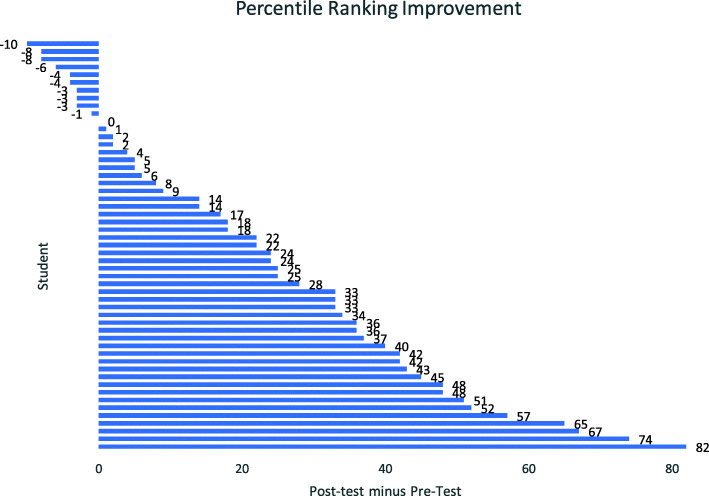
Percentile difference scores

Pre-test percentile scores for students in the study university averaged 58.7 (median of 63). For the post-test, the average jumped to 80.44 (median of 84). The mean percentile increase was 21.7 percentile points, a significant jump. Figure 7 depicts the individual percentile difference scores.

Analysis of post-test results against other universities was revealing. The study university was compared to other SACS-accredited bodies as well as those accredited by the Higher Learning Commission (HLC). Also, the study university was compared against hybrid, online and traditional campuses. Table 2 provides the results overall and by competency area (*n* = 119 post-tests). The utility of such analysis is that it informs performance improvement.

**Table 2:** Comparison of study university scores versus other universities using PAS

Competency	Score	SACS	HLC	Hybrid	Online	Traditional
Total	71.51	58.71	60.87	57.88	63.99	58.66
Financial Management	73.52	51.65	53.41	51.45	56.98	53.53
General Management	77.22	58.83	60.19	58.79	67.45	59.78
Healthcare Personnel	79.44			66.10	66.82	65.35
Healthcare Systems and Organizations	67.96	57.17	59.93	55.70	62.22	57.06
Information Management	66.66	54.45	56.66	53.15	54.44	53.84
Leadership Skills and Behavior	77.04				68.78	
Managing Change	70.56					
Organizational Climate and Culture	75.74					
Quality Improvement	72.96		61.49	54.79	61.67	56.32
Strategic Planning and Marketing	77.41	55.53	57.76	55.40	64.46	57.30
The Community and the Environment	68.15					52.06
The Legal Environment	74.63	53.36	55.47	51.43		54.73

### Inferential statistics

Paired sample t-tests were run for the final score and each competency separately. To account for familywise error, the *p*-values were adjusted via Holm’s method. In all cases, the findings were statistically significant at the .05 level; there was evidence that student performance improved. Table 3 provides the estimates, the 95% confidence intervals, the t-values, and the Holm-adjusted *p*-values.

**Table 3:** Paired t-tests

Competency Area	Estimate	Lower 95% CI	Upper 95% CI	***t***-Value	df	Holm-Adjusted ***p***-value
Strategic Planning and Marketing	21.296	15.888	26.705	7.898	53.000	<.001
Leadership Skills and Behavior	18.333	12.604	24.062	6.418	53.000	<.001
General Management	17.963	12.292	23.634	6.354	53.000	<.001
Financial Management	17.593	11.433	23.752	5.729	53.000	<.001
Quality Improvement	15.000	9.420	20.580	5.392	53.000	<.001
The Community and the Environment	15.000	8.754	21.246	4.817	53.000	<.001
Healthcare Personnel	14.815	9.037	20.593	5.143	53.000	<.001
Healthcare Systems and Organizations	14.630	7.443	21.816	4.083	53.000	<.001
The Legal Environment	13.519	7.021	20.016	4.173	53.000	<.001
Organizational Climate and Culture	11.852	6.548	17.156	4.482	53.000	<.001
Managing Change	9.074	3.738	14.410	3.411	53.000	0.001
Information Management	8.333	2.340	14.326	2.789	53.000	0.007
Total Score	12.851	10.521	15.181	11.062	53.000	<.001
Percentile Rank	21.704	14.755	28.652	6.265	53.000	<.001

After failing to reject the null assumption of multivariate normality via Mardia’s test [33], MANOVA models of the difference in subscores were evaluated as a function of gender, ethnicity, and a dichotomously coded grade-point average variable (0 = < 3.0, 1 = ≥3.0). Results of the analysis show that only the intercept is significant in evaluating difference scores for the competencies. GPA, gender, and ethnicity did not affect performance. See Table 4 for the results. Additional analysis is available here: https://rpubs.com/R-Minator/PASTesting.

**Table 4:** MANOVA Results, DF = Degrees of Freedom

Term	Df	Pillai’s Trace	Approximate ***F***-Value	Numerator DF	Denominator DF	***p***-value
(Intercept)	1	0.8086	13.0292	12	37	<.001
Gender	1	0.2096	0.8175	12	37	0.63160
Ethnicity	3	0.6581	0.9132	36	117	0.61220
GPA	1	0.1278	0.4519	12	37	0.92960

## Discussion

### Summary of findings

The primary research question investigated whether pre-post, third-party objective testing provided evidence that a medical education program effectively increases one or more of the 12 student competencies assessed. The results confirm that all competencies were improved through the program curriculum intervention as would be expected. This type of improvement is suggestive of some face validity. The “average” student increased approximately 13 points (from 59 to 72), and mean post-test scores were statistically better than the pre-test scores in all competency areas with *p* < .001. In comparison to other universities’ post-tests, the study university’s average scores of 71.5 are higher than any of the comparative groups: SACS programs, HLC programs, hybrid programs, online programs, traditional programs. The ability to compare across programs allows for benchmarking.

In the study program, all evaluated competency areas improved significantly. The largest raw score improvement was in strategic management (21.3 points). The smallest improvement was in health information management (8.3 points). This information was used as part of program performance improvement to review and revamp the Health Information Management course. While testing is certainly not the panacea for program assessment, it did identify an area for improvement.

Multivariate models of the difference scores resulted in no statistically significant associations with GPA, ethnicity, gender, or age. Only the differences themselves (e.g., the performance improvement from pre-test to post-test) remain statistically significant. For many medical education programs that serve ethnically and racially diverse populations, evaluating test effects by diversity is something that must be considered.

### Comparison with other assessment options

To date, various competency assessments and related competency models have been developed and utilized to validate HAML programs [7, 12, 21, 34–36]. Prior to the switch to PAS evaluation, the program’s exit exam initiatives were conducted at an internal level with varying methods of individual student assessment and related analyses (Lieneck, 2011). None of these assessments was based on third-party objective testing, so PAS may be an improvement.

Many university programs have opted to create their own internal method of competency assessments (some to include both pre-post assessments). The use of non-standardized assessments results in ambiguity of competency assessment terminology and methodological deployment [37]. Furthermore, with regard to academic competency evaluation in healthcare administration students, many faculty charged with developing healthcare administration program exit examinations are not well trained in the art of measurement and the science of testing [37]. PAS testing in this study has shown a program curriculum effect, which suggests that the evaluation has some prima facia validity. Further, several BHA faculty took the examination to ensure the test evaluated appropriate topic areas in a reasonable fashion and that, in general, the faculty were satisfied with their experiences.

### Importance for medical educators

There are several important considerations for medical educators. First, multiple methods and multiple assessments are necessary for competency evaluation [38]. One single assessment cannot assess program-wide competency goals. Second, when assessing competencies, leaders need to evaluate program metrics over time. These repeated measures help assess reliability of the program and control for the largest variance producer in at least one study, the student [39]. Without such a baseline and repeated measurements, it is impossible to gauge the effectiveness of teaching on competency attainment. Third, national and international standards for competency attainment should be sought for programs that currently have none, similar to the efforts of the United States and Europe to reshape the future of dental education [40]. Without these standards, it is impossible to gauge the performance of students relative to national and international standards. Seeking out methods for external assessment of competencies becomes increasingly important given these facts. Finally, medical educators have the responsible to assess their contributions to competency building using methods that are robust and replicable, regardless of field. A perfect example outside this study is the measurement of competencies conducted by the Faculty of Veterinary Medicine at Utrecht University in the Netherlands [39].

### Limitations

While HAML certifying and accrediting bodies (AUPHA and CAHME) coupled with educational institutions are assisting PAS in building a repository of questions that demonstrate content validity in assessing competencies beyond the knowledge level of Bloom’s taxonomy, there are many associated with PAS testing. First, PAS is not structured based on computer adaptive testing (CAT) such as the Graduate Record Examination (GRE). Computer adaptive testing results in greater achievement measurement precision and improved student motivation and engagement [41]. Since questions are not coded by difficulty (another limitation), there is no mechanism for assessing students’ true competency capability through algorithmic assessment. Instead, the competency capability is assessed as the percentage correct attained during testing. This issue has been addressed with PAS by the program; however, it is a major limitation in the quality of the examination administration.

Another limitation of PAS is the inability of faculty to review the questions in the databank. The questions are under review through AUPHA experts, but the end date for that review is unknown. Understandably, PAS would like to keep this information proprietary; however, doing so requires that students keep records of questions which area questionable.

A third limitation is induced incentive bias. Since the pre-test is not graded, students have little incentive to perform well other than internal motivation. Reasonable performance on the post-testis necessary for students to proceed to the residency, though.

The questions of PAS often are knowledge rather than pure competency based, an added limitation. While some questions require students to apply acquired knowledge, many require only a base knowledge level. Further, the PAS pre-packaged health administration competencies do not include quantitative skills, so the study program had to request the use of business school competencies for assessment.

The PAS questions are unable to assess competencies such as communication skills and leadership. “Soft skills,” the ones sought by many organizations when making management hiring decisions, are not well-assessed by examinations of this nature.

Despite these limitations, the results show some prima facia validity in that one would expect an educational intervention to result in improved performance on a test designed to measure competencies in the program area. The limitations in the PAS testing process are to be addressed as part of process improvement; however, PAS in its current state does provide a third-party, objective measure for evaluating competencies.

## Conclusions

The PAS healthcare administration examination is relatively new to AUPHA and the program under study. Performance improvement requires continuous assessment of the examination and its ability to measure competencies effectively. Ongoing analysis of exam results will continue to provide valuable program and industry-level information to healthcare administration education stakeholders. Programs investing in this external exam are encouraged to perform their own program-level analyses, as well as the potential for valuable inter-program collaboration to best improve the development of future healthcare leaders.

This study is ongoing, and analysis is conducted every semester based on the outcomes. If PAS improves and adds computer adaptive testing, programs using this third-party tool may benefit from increased student satisfaction and achievement based on previous research [41]. Further, programs may use the results for quality improvement, and they may benchmark program performance against other universities. In this case, however, there are many validity issues that should be addressed for widespread use of this one element of competency assessment.

## Data Availability

The anonymous datasets used during and/or analyzed during the current study available from the corresponding author on reasonable request and approval by the IRB. A complete list of program and student learning objective assessments (as well as the analysis) is available online: https://rpubs.com/R-Minator/PASTesting.

## Abbreviations

AUPHA: Association of University Programs in Health Administration
BHA: Bachelor of Healthcare Administration
CAHME: Commission on Accreditation of Healthcare Management Education
CBME: Competency-based medical education
GPA: Grade Point Average
HAML: Healthcare administration, management, and leadership
HIM: Health Information Management
PAS: Peregrine Academic Services
